# Mitochonic acid 5 increases boar sperm quality by mitigating mitochondrial dysfunction

**DOI:** 10.3389/fcell.2025.1583951

**Published:** 2025-06-16

**Authors:** Ruyuan Wang, Adedeji O. Adetunji, Lingjiang Min, Zhendong Zhu

**Affiliations:** ^1^ College of Animal Science and Technology, Qingdao Agricultural University, Qingdao, China; ^2^ Department of Agriculture, University of Arkansas at Pine Bluff, Pine Bluff, AR, United States

**Keywords:** boar sperm, quality, mitochondrial, metabolism, mitochonic acid 5

## Abstract

Mitochondrial dysfunction causes an increase in oxidative stress and depletion in ATP production. Studies have shown that mitochonic acid 5 (MA-5) improves cellular mitochondrial metabolism and ATP production. To this end, varying concentrations of MA-5 (0.01, 0.1, 1, and 10 nM) were added to a BTS extender, and thereafter, sperm motility and morphological parameters were assessed. Results indicate that adding 1 nM MA-5 significantly enhanced boar sperm motility and progressive motility, with higher values observed at the 1- and 2-h time points compared to the control. Sperm membrane and acrosome integrity values between the control and treatment groups were not different, except for 10 nM MA-5, which caused a reduction in membrane integrity. Treatment with MA-5, specifically 1 nM MA-5, also significantly boosted mitochondrial membrane potential and ATP content at the 2-h incubation point. In addition, MA-5 treatments stabilized mitochondrial transcription and translation processes, maintained overall sperm functionality, increased NADPH dehydrogenase subunits 1 (MT-ND1) and NADPH dehydrogenase subunits 6 (MT-ND6) protein and gene expression, and affected mitochondrial transcription factor A (TFAM) levels. However, after 4 h of incubation with MA-5, a decline in sperm quality parameters and an increase in ROS levels were observed. Interestingly, adding 10 nM pyrroloquinoline quinone (PQQ) and MA-5 to the extender restored mitochondrial function by enhancing mitochondrial potential and ATP content after 4 h of incubation. Overall, treatment with 1 nM MA-5 can help maintain sperm quality and mitochondrial metabolism during incubation (up to 2 h) at 37^o^C. Moreover, addition of a combination of MA-5 and 10 nM PQQ to the boar sperm extender is crucial for maintaining high-quality reproduction after 4 h of incubation. Consequently, MA-5 and PQQ play a role in maintaining sperm quality and mitochondrial function and can help in boar sperm preservation and artificial insemination (AI) practices.

## 1 Introduction

The success of the swine breeding industry in recent years can be attributed to notable improvements in genetic indices, improved fertility, and the adoption of efficient artificial insemination (AI) practices (Knox, 2014). The optimization of semen extenders in AI is crucial for obtaining high-quality reproductive performance in boars (Galić et al., 2022). To ensure sperm viability, successful fertilization, and optimal conception rates, sperm linear motility must be maintained during semen transport and preservation. In addition, linear motility, defined as low lateral amplitude and high straight-line velocity, is essential for sperm migration from the cervix to the uterus and then to the oviduct (Shalgi et al., 1992; Suarez and Pacey, 2006).

The mitochondria play a pivotal role in governing various sperm functions, including motility and capacitation (Guthrie et al., 2008; Koppers et al., 2008; Guthrie and Welch, 2012; Gibb et al., 2014; Moraes and Meyers, 2018). Although the mitochondria are the major source of reactive oxygen species (ROS) in the sperm (Aitken, 2020), studies have shown that NADPH oxidase 5 (NOX5), aromatic amino acid oxidases, and lipoxygenases are other sources of ROS (Musset et al., 2012; Aitken, 2017). However, elevated ROS level-induced oxidative damage can be caused by any disruption in the mitochondrial electron transport chain (Murphy, 2016). This damage compromises sperm quality, impairing DNA integrity, membrane stability, and other critical functions, including adenosine triphosphate (ATP) production, motility, and capacitation. According to Rodríguez-Enríquez et al. (2010), the mitochondria account for 79% of cellular ATP production in cervical carcinoma HeLa cells and 91% in breast carcinoma MCF cells (Chang et al., 2019).

Energy is required to maintain viability, promote proliferation, and fulfill physiological functions in mammalian cells (Rodríguez-Enríquez et al., 2010). Similarly, to generate energy for cellular functions such as motility, capacitation, and interaction with female oocytes, sperm utilize a variety of metabolic substrates (Rodriguez‐Gil, 2006; Rodríguez-Gil and Bonet, 2016; Moraes and Meyers, 2018). Numerous studies have investigated the metabolic strategies of sperm, specifically focusing on the role of the mitochondria in the energy supply system (Peña et al., 2015; Plaza Davila et al., 2015; Davila et al., 2016). This movement considerably relies on adenosine triphosphate molecules, which serve as the primary energy carrier in cells and are crucial for maintaining cellular functions (Folgerø et al., 1993). ATP production mainly occurs through two distinct processes. One of these processes is OXPHOS, which occurs in the mitochondria, and the other is glycolysis, which takes place in the cytosol of the cell, working in tandem to maintain energy balance in response to microenvironmental fluctuations (Calamera et al., 2009; Calamera et al., 2010; Grüning et al., 2011; Du-Plessis et al., 2015; Zhu et al., 2019a). The relative contribution of these pathways to ATP production depends on the type of cell, its stage of growth, and environmental conditions (Galantino-Homer et al., 2006; Zhu et al., 2019a).

Mitochonic acid-5 (MA-5), a derivative of the plant hormone indole-3-acetic acid, acts as an accelerator, enhancing ATP synthesis (Suzuki et al., 2015; Wang et al., 2024). Recent studies demonstrate that MA-5 enhances mitochondrial function by boosting mitochondrial energy metabolism in fibroblasts (Ohwada et al., 2008; Zhang et al., 2014). Additionally, our previous study on rams indicated that MA-5 supplemented in the tris-citrate-glucose extender maintained sperm mitochondria function when stored at 4°C (Wang et al., 2024). Although the use of antioxidants such as pyrroloquinoline quinone (PQQ) has been shown to reduce mitochondrial damage and improve boar sperm linear motility by enhancing mitochondrial ATP production (Zhu et al., 2019a), the effects of MA-5 as a diluent for semen extenders in boar sperm have not yet been elucidated. To this end, this study hypothesized that adding MA-5 to a sperm extender could enhance the quality of boar sperm by stimulating the mitochondrial function in order to improve the pregnancy rate for AI and the number of births.

## 2 Materials and methods

### 2.1 Chemicals

All chemicals and reagents, unless otherwise stated, were obtained from Sigma-Aldrich® Agricultural Technology Development Co., Ltd. (Shanghai, China). Mitochonic acid 5 (Cat. No. S0881), 4-(2,4-difluorophenyl)-2-(1H-indole-3-yl)-4-oxobutanoic acid, was obtained from Selleck Chem (China). Pyrroloquinoline quinone disodium salt (PQQ) was procured from MedChemExpress Co., Inc. (Monmouth Junction, NJ, United States). MA-5 was initially solubilized in 0.01% DMSO to prepare the stock solution.

### 2.2 Ethical approval

All animals and experimental procedures were approved by the Qingdao Agriculture University Institutional Animal Care and Use Committee (QAU1121010).

### 2.3 Boar semen collection and processing

For this study, six healthy, fertile, and mature Duroc boars were selected, and they were subjected to three consecutive weekly semen collections by use of the gloved hand technique. Each boar was housed separately in natural daylight conditions and fed basal diets with unrestricted access to water. A double gauze was used to filter the sperm-rich fraction of the semen collected before analysis. In total, 54 ejaculates were obtained from the six boars, and each of the ejaculates was kept separately during the transit to the laboratory. The assessment of the ejaculated semen motility was done using a computer-assisted sperm analysis (CASA) system, imposing a rigorous inclusion criterion demanding over 90% motility in the semen samples (Umehara et al., 2018). Furthermore, the semen obtained from the six boars was pooled and divided into five aliquots to reduce individual variations. First, 1 mL each from five groups of sperm (30 million/mL) was incubated with various concentrations of MA-5 (0.01, 0.1, 1, and 10 nM) dissolved in 0.1% DMSO for 1, 2, and 4 h at 37^°^C. Second, 1 nM MA-5 and 10 nM PQQ were incubated with two groups of sperm (30 million/mL) for up to 4 h. The BTS extender is composed of 12.8 mM trisodium citrate, 14.3 mM NaHCO_3_, 9.9 mM EDTA·2Na, 26.7 mM citric acid, 1,000 U/mL penicillin G potassium salt, 1 mg/mL streptomycin sulfate, 0.1 mg/mL polymyxin B sulfate, and 330 mM glucose.

### 2.4 Evaluation of sperm motility using a computer-assisted sperm analysis (CASA) system

A computer-assisted sperm analysis (CASA) system (HT CASA-Ceros II; Hamilton, MA, United States) was used to assess sperm motility, and sperm trajectories (0.5 s, 45 frames) were systematically captured at 60 Hz. Overall, more than 200 individual trajectories were recorded. The sperm motility parameters measured include straight-line velocity (VSL, µm/s), curvilinear velocity (VCL, µm/s), average path velocity (VAP, µm/s), linearity (LIN, %), straightness (STR, %), wobble (WOB, %), total motility (%), and progressive motility (%). VCL, LIN, VAP, VSL, STR, and WOB were calculated for only motile sperm. Before sample evaluation on CASA, 5 µL of each sperm sample was dispensed into a prewarmed Makler sperm-counting chamber (10 µm depth; Haifa Instruments, Haifa, Israel), as previously described by Zhu et al. (2019a).

### 2.5 Evaluation of sperm membrane integrity and acrosome integrity

Membrane integrity of sperm was determined using LIVE/DEAD™ Sperm Viability Kit (L711, Thermo Fisher, Shanghai, China) (Zhang et al., 2022). Hundred microliters of the sperm sample was first mixed with 0.1 µL of SYBR-14 and 0.5 µL of propidium iodide (PI) (Zhang et al., 2022). Thereafter, the mixture was incubated at 37°C for 10 min in the dark. After staining, the sperm were visualized with the aid of a fluorescence microscope (ZEISS DM200LED, Oberkochen, Germany). The procedure led to the selective labeling of dead (sperm with plasma membrane damage, red arrow, Supplementary Figure S1) and live (sperm with intact membrane, white arrow, Supplementary Figure S1) sperm cells as red or green fluorescence, respectively. At least 200 sperm were evaluated and scored for each treatment sample.

Sperm acrosome integrity evaluation was performed using fluorescein isothiocyanate peanut agglutinin (FITC-PNA, L7381, Sigma, Shanghai, China) and PI (Zhang et al., 2022). Thirty microliters of sperm was placed on a slide and fixed in 75% methanol. Thereafter, the fixed sperm sample was then incubated at 37°C for 30 min in the dark after adding 30 µL of an FITC-PNA solution. The sample was incubated again for 10 min with another 20 µL of the PI solution. Thereafter, acrosome integrity was detected using a fluorescence microscope (ZEISS DM200LED). As shown in Supplementary Figure S2, there are two groups of sperm after staining with FITC-PNA/PI: damaged acrosome (red arrow) and intact acrosome (white arrow). At least 200 sperm were evaluated and scored for each treatment sample.

### 2.6 Mitochondrial membrane potential

The sperm mitochondrial membrane potential was evaluated using a JC-1 Assay Kit (911, ImMunoChemistry Technologies, LLC, Davis, CA, United States) (Zhu et al., 2022). Sperm samples were incubated with 500 µL of 1× working solution for 30 min at 37°C in the dark. Thereafter, flow cytometry comprising a filter with a bandwidth of 574/26 nm (AttuneOR NxT Acoustic Focusing Cytometer, FACSCalibur, BD Biosciences, Carlsbad, CA, United States) was used to evaluate sperm mitochondrial membrane potential. Sperm mitochondrial activity was calculated as the mean fluorescence intensity (MFI) of JC-1 aggregates; 10,000 sperm events were analyzed.

### 2.7 Intracellular sperm mitochondrial ROS level

Intracellular sperm mitochondrial ROS level was assessed using a MitoSOX™ Red Assay Kit (M36008, Thermo Fisher Scientific K.K., Shanghai, China) (Zhu et al., 2022) based on the manufacturer’s instructions. Measurements were expressed as the mean fluorescence intensity (MFI). Sperm samples were incubated for 10 min at 37°C in the dark with a working solution of 500 µL of MitoSOX™ Red reagent (5 µM). Thereafter, flow cytometry comprising a filter with a bandwidth of 574/26 nm was used to evaluate mitochondrial ROS levels, and 20,000 sperm-specific events were analyzed.

### 2.8 Sperm ATP and malondialdehyde (MDA) levels

An Enzylight™ ATP Assay Kit (EATP100, Bioassay System, Hayward, CA, United States) was used to determine sperm ATP contents (Birben et al., 2012). According to the manufacturer’s instructions, the substrate and assay buffer were mixed with sperm samples. Thereafter, ATP content was measured using a luminometer (2030 Multilabel Reader ARVO X4; PerkinElmer Inc., Waltham, MA, United States).

An MDA Assay Kit (Jian Cheng Bioengineering Institute, Nanjing, China) was used to assess sperm MDA contents (Zhu et al., 2024). To this end, the experimental buffer and substrate were incubated with sperm samples. Thereafter, analysis was conducted using a spectrophotometer (2030 Multilabel Reader ARVO X4; PerkinElmer Inc., Waltham, MA, United States).

### 2.9 Western blotting

Total sperm protein extraction was performed using sodium dodecyl sulfate (SDS) sample buffer based on a previous study by Zhu et al. (2022). Protein separation was carried out on a 12.5% SDS-polyacrylamide gel electrophoresis (SDS-PAGE), and the separated proteins were then transferred to a polyvinylidene difluoride (PVDF) membrane (GE Bioscience, Newark, NJ, United States). Nonspecific binding was prevented by incubating the membranes in Tris-buffered saline (TBS) containing 0.1% (v/v) Tween-20% and 5% (w/v) bovine serum albumin (Life Technologies, Grand Island, NY, United States). The following primary antibodies, anti-mitochondrial NADPH dehydrogenase subunit 1 (anti-MT-ND1; 1973-1-AP, Proteintech, Rosemont, IL, United States), anti-mitochondrial NADPH dehydrogenase subunit 6 (anti-MT-ND6; bs-3955R; Bioss, Inc., Boston, MA, United States), and anti-α-tubulin (2148; Cell Signaling Technology, Inc., Danvers, MA, United States), were diluted at a ratio of 1:1,000 in 5% bovine serum albumin in TBS–Tween and incubated overnight at 4°C. Thereafter, the PVDF membranes were placed in a TBST solution for washing. Following this, the membranes were incubated with HRP-conjugated secondary antibodies (goat anti-rabbit for MT-ND1, MT-ND6, and goat anti-mouse α-tubulin) at a 1:5,000 dilution for 1 h. The membrane was washed in TBST, and then enhanced chemiluminescence (ECL) substrate was applied for detection. The band intensities were quantified with a Gel-Pro Analyzer (Media Cybernetics, Rockville, MD, United States).

### 2.10 Co-immunoprecipitation (Co-IP)

Sperm proteins were extracted using lysis buffer (supplemented with protease inhibitor cocktail, Roche, Indianapolis, IN, United States) at 4°C for 30 min, followed by centrifugation at 12,000 × g for 30 min. The supernatant was divided for subsequent analyses: one aliquot was reserved for Western blotting, while another was incubated overnight at 4°C with primary antibodies against 4-HNE (1:20 dilution) or POLRMT (1:20 dilution). Protein G agarose beads (5873S, Cell Signaling Technology; 1:20 dilution) were then added to the sperm lysate and gently rotated at 4°C for 3 h. After five washes with lysis buffer, the immunoprecipitated complexes were resuspended in 20 μL of 1× SDS sample buffer and subjected to Western blotting analysis (Zhu et al., 2019a).

### 2.11 RNA extraction, reverse transcription, and quantitative PCR analyses

Quantitative PCR analysis was conducted as described previously (Shimada et al., 2008). To extract sperm, the total RNeasy mini kit (QIAGEN Sciences, Germantown, MD, United States) was utilized. To perform reverse transcription (RT), 500 ng polydeoxythymidine and 0.25 U avian myeloblasts virus reverse transcriptase (Promega, Madison, WI, United States) were used at 42°C for 75 min and at 95°C for 5 min. Thereafter, cDNA and specific primers listed in Table 1 were used to carry out quantitative real-time PCR analyses, as described previously (Zhu et al., 2019b).

**TABLE 1:** List of primers and their sizes.

Gene name	Primer sequence	Product size (kb)
*MT-ND1*	F: AATATGGCGAAAGGTCCGGC	104
	R: ACCCTAGCAGAAACCAACCG	
*MT-ND6*	F: AAGCAGCAATCCCCATAGCTT	118
	R: GCGTTGAAGGAAGAGGAAGTAGA	

*MT-ND1*, NADPH dehydrogenase subunits 1; *MT-ND6*, NADPH dehydrogenase subunits 6.

### 2.12 Statistical analysis

Statistical comparisons across three replicates were conducted using one-way analysis of variance (ANOVA), followed by Duncan’s new multiple range test (95% confidence interval). The data obtained were expressed as mean ± standard deviation (SD) values. P < 0.05 was considered statistically significant.

## 3 Results

### 3.1 MA-5 improves sperm quality

**FIGURE 1:**
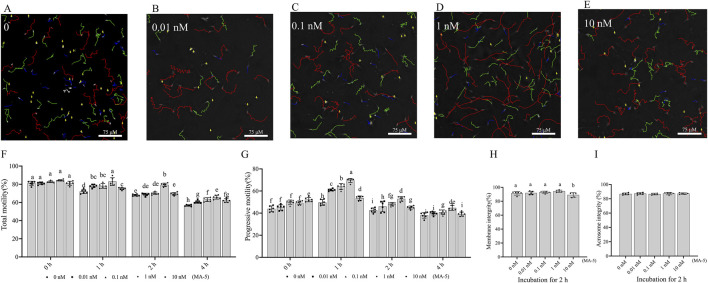
Effects of MA-5 on sperm motility, membrane integrity, and acrosome integrity during incubation at 37°C. **(A–G)** Sperm motility was assessed using computer-assisted sperm analysis (CASA) following incubation with MA-5 for 4 h (n = 6). **(A–E)** Changes in the motility tracks of sperm generated by the CASA system at the 2-h time point of incubation. Total motility **(F)** and progressive motility **(G)** of boar sperm incubated with MA-5 for 4 h at concentrations ranging from 0 to 10 nM (n = 6). Membrane integrity **(H)** and acrosome integrity **(I)** were assessed after 2 h of incubation with MA-5 at concentrations of 0–10 nM (n = 5). Values are specified as mean ± standard deviation (SD). Columns with different lowercase letters indicate significant differences (p < 0.05). Bars = 75 μm. MA-5: mitochonic acid 5.

The CASA system-generated sperm tracks depicted in Figures 1A–E showed a significant enhancement (p < 0.05) in boar sperm motility patterns with the addition of various concentrations of MA-5 during incubation. Results from CASA also indicated that the motility of boar sperm treated with 1 nM MA-5 was higher than that of the control at 1- and 2-h time points. Notably, the MA-5 treatment decreased sperm progressive motility at the 4-h incubation time point compared to those at 1- and 2-h time points. In addition, 1 nM MA-5 treatment yielded the highest progressive motility values at 1- and 2-h time points (Figures 1F,G). Furthermore, at the 1- and 2-h time points of incubation, supplementation with 1 nM MA-5 in BTS medium resulted in a significant increase in parameters such as VCL, VSL, VAP, and STR, whereas the LIN, WOB, ALH, and BCF showed no significant difference compared to the control (Table 2; Supplementary Figure S8).

**TABLE 2:** Effects of different concentrations of MA-5 on motility parameters of boar sperm.

Different of concentrations of MA-5 (nM)						
Sperm parameters	Time (h)	0	0.01	0.1	1	10
VCL (μm/s)	1	32.15 ± 3.05^bc^	35.71 ± 1.00^b^	37.60 ± 2.30^a^	38.74 ± 1.89^a^	30.07 ± 0.68^c^
	2	39.51 ± 1.99^b^	44.82 ± 5.30^ab^	44.30 ± 0.58^a^	39.51 ± 1.33^b^	34.18 ± 0.37^c^
	4	50.36 ± 4.21^a^	45.77 ± 1.32^a^	47.19 ± 3.39^a^	45.83 ± 2.05^a^	39.81 ± 6.07^b^
VSL (μm/s)	1	24.20 ± 2.50^c^	26.31 ± 0.83^b^	27.30 ± 0.67^b^	29.14 ± 1.71^a^	20.84 ± 2.07^days^
	2	27.83 ± 1.99^b^	32.52 ± 0.82^a^	34.59 ± 2.10^a^	33.94 ± 1.50^a^	24.39 ± 0.17^c^
	4	28.44 ± 0.85^a^	28.54 ± 1.96^a^	27.74 ± 2.88^a^	28.92 ± 0.81^b^	25.96 ± 0.68^b^
VAP (μm/s)	1	28.21 ± 2.52^bc^	30.69 ± 0.84^b^	32.26 ± 3.43^a^	34.21 ± 1.64^a^	24.96 ± 2.03^c^
	2	34.94 ± 1.71^b^	39.32 ± 1.13^a^	40.58 ± 2.90^a^	39.25 ± 1.72^a^	28.79 ± 0.32^c^
	4	35.89 ± 3.36	34.72 ± 2.80	35.76 ± 0.76	33.68 ± 0.89	34.74 ± 4.98
LIN (%)	1	75.21 ± 0.63	73.68 ± 2.05	73.64 ± 3.30	75.20 ± 0.75	69.41 ± 8.27
	2	72.03 ± 1.55	71.04 ± 0.38	73.33 ± 0.89	74.06 ± 0.25	71.36 ± 0.28
	4	55.30 ± 1.22^c^	63.93 ± 3.18^b^	61.64 ± 2.10^b^	73.21 ± 0.51^a^	66.03 ± 8.01^a^
STR (%)	1	85.71 ± 1.15	85.72 ± 1.25	86.01 ± 0.30	85.16 ± 0.90	83.41 ± 1.79
	2	81.45 ± 1.56^b^	82.71 ± 0.30^b^	85.28 ± 0.98^a^	85.87 ± 0.14^a^	84.74 ± 0.36^a^
	4	77.63 ± 1.84^b^	82.26 ± 1.00^a^	76.34 ± 0.50^b^	82.48 ± 0.11^a^	75.54 ± 8.41^ab^
WOB (%)	1	88.43 ± 0.43	85.90 ± 0.31	85.63 ± 4.09	88.30 ± 0.07	83.11 ± 8.36
	2	87.76 ± 0.45	85.95 ± 1.15	85.98 ± 0.05	85.64 ± 0.19	84.21 ± 0.11
	4	71.26 ± 2.34^b^	77.69 ± 2.93^b^	80.74 ± 2.36^b^	85.26 ± 0.72^a^	87.36 ± 1.11^a^
ALH (μM)	1	2.13 ± 0.14	2.22 ± 0.08	2.49 ± 0.40	2.56 ± 0.09	2.15 ± 0.26
	2	2.24 ± 0.06	2.47 ± 0.05	2.78 ± 0.05	2.34 ± 0.09	2.77 ± 0.04
	4	2.82 ± 0.41	2.85 ± 0.22	2.90 ± 0.12	2.85 ± 0.06	2.18 ± 0.20
BCF (Hz)	1	4.71 ± 0.08	4.78 ± 0.19	4.41 ± 0.02	4.16 ± 0.06	4.72 ± 0.19
	2	4.66 ± 0.06	4.73 ± 0.04	4.20 ± 0.06	4.85 ± 0.13	3.96 ± 0.07
	4	5.11 ± 1.01	4.27 ± 0.07	4.56 ± 0.85	4.24 ± 0.03	4.50 ± 0.44

Parameters include curvilinear velocity (VCL), straight-line velocity (VSL), average path velocity (VAP), linearity (LIN; VSL/VCL), straightness (STR; VSL/VAP), wobble (WOB; VAP/VCL), amplitude of lateral head displacement (ALH), and beat-cross frequency (BCF) (Hz) (n = 6).

In addition, the addition of different concentrations (0.01–1 nM MA-5 treatment) did not significantly change (p > 0.05) membrane integrity compared to that of the control during the 2-h incubation period. However, the 10 nM MA-5 treatment decreased sperm membrane integrity (Figure 1H). In addition, the addition of various concentrations of MA-5 also did not significantly affect sperm acrosome integrity after 2 h of incubation (Figure 1I, p > 0.05).

### 3.2 MA-5 enhances sperm mitochondrial membrane potential and ATP content and increases ROS levels and MDA content

**FIGURE 2:**
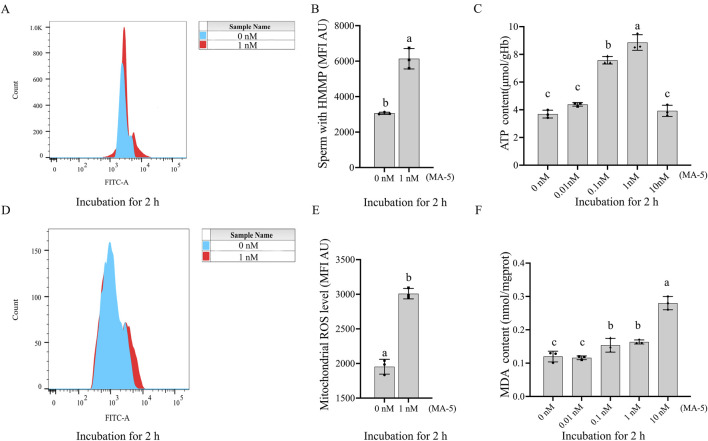
Effects of MA-5 on mitochondrial membrane potential, ATP production, ROS levels, and lipid peroxidation in boar sperm at the 2-h time point of incubation. **(A, B)** Flow cytometry analysis of mitochondrial membrane potential in boar sperm after treatment with 0 nM and 1 nM MA-5 using JC-1 staining. **(C)** ATP levels in boar sperm after exposure to 0, 0.01, 0.1, 1, and 10 nM MA-5. **(D, E)** Flow cytometric analysis of mitochondrial ROS levels in boar sperm after treatment with 0 nM and 1 nM MA-5. **(F)** Malondialdehyde (MDA) levels in boar sperm following exposure to 0, 0.01, 0.1, 1, and 10 nM MA-5. Values are specified as means ± standard deviation (SD). Columns with different lowercase letters indicate significant differences (p < 0.05) (n = 3). HMMP: sperm with high mitochondrial membrane potential. ROS: reactive oxidative species.

The mitochondrial membrane potential of boar sperm in both the control and 1 nM MA-5 treatment groups was analyzed after 2 h of incubation at 37°C. The addition of 1 nM MA-5 significantly increased the mitochondrial membrane potential compared to the control in the BTS extender (Figures 2A,B, p < 0.05).

The addition MA-5 at concentrations ranging from 0.1 nM to 1 nM significantly increased (p < 0.05) sperm ATP content compared to the control. Analysis revealed that the addition of MA-5 at 1 nM significantly increased sperm ATP content compared to the control after 2 h in the BTS extender (Figure 2C, p < 0.05).

In addition, the supplementation of 1 nM MA-5 significantly increased ROS levels compared to the control (Figures 2D, E, p < 0.05). Similarly, the supplementation of MA-5 increased MDA content compared to the control at 2 h, except for 0.01 nM MA-5 (Figure 2F, p < 0.05). Treatment with 1 nM MA-5 increased the sperm ROS level and MDA level, which might be due to the high mitochondrial membrane potential activated by the MA-5.

### 3.3 MA-5 enhanced boar sperm mitochondrial transcription and translation

**FIGURE 3:**
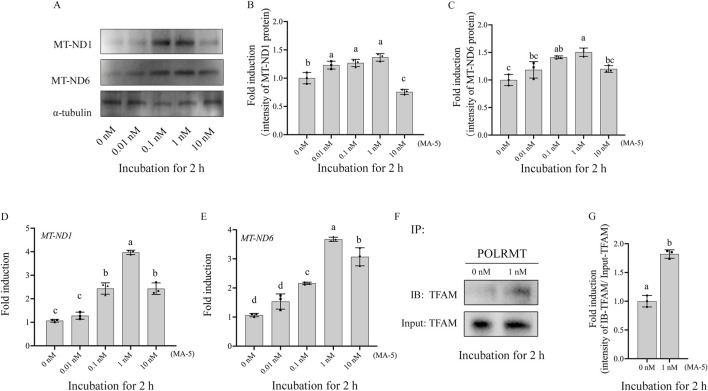
MA-5 enhances mitochondrial function in boar sperm by regulating MT-ND1, MT-ND6, and TFAM–POLRMT complex protein expression at the 2-h time point of incubation. **(A–C)** Western blotting analysis of MT-ND1 and MT-ND6 in boar sperm. **(D, E)** Expression of *MT-ND1* and *MT-ND6* genes after treatment with MA-5 for 2-h incubation. **(F)** Immunoprecipitates were immunoblotted (IB) with TFAM antibody. **(G)** Quantitative expression of IB-TFAM over the input generated from Western blotting. MT-ND1: NADPH dehydrogenase subunits 1; MT-ND6: NADPH dehydrogenase subunits 6; TFAM: mitochondrial transcription factor A; POLRMT: mitochondrial RNA polymerase. Values are specified as means ± standard deviation (SD). Columns with different lowercase letters indicate significant differences (p < 0.05) (n = 3).

The expression of mitochondrial proteins (MT-ND1 and MT-ND6) in boar sperm after being treated with different doses of MA-5 was analyzed after 2 h of incubation at 37°C (Figures 3A–C; Supplementary Figure S3). Addition of 1 nM of MA-5 to the BTS medium significantly increased the expression of mitochondrial proteins in boar sperm compared to that in the control (Figures 3A–C, p < 0.05). In addition, after incubation for 2 h, the expression levels of *MT-ND1* and *MT-ND6* RNA were also significantly increased compared with those of the control (Figures 3D, E, p < 0.05). Furthermore, after 2-h incubation with MA-5 in a BTS medium, the sperm POLRMT and TFAM proteins formed a complex, and their levels were significantly increased with 1 nM MA-5 treatment (Figures 3F,G; Supplementary Figure S4, p < 0.05).

### 3.4 Addition of PQQ to the MA-5 treatment enhanced sperm motility, mitochondrial membrane potential, and ATP content and reduced ROS level and MDA content at 4 h

**FIGURE 4:**
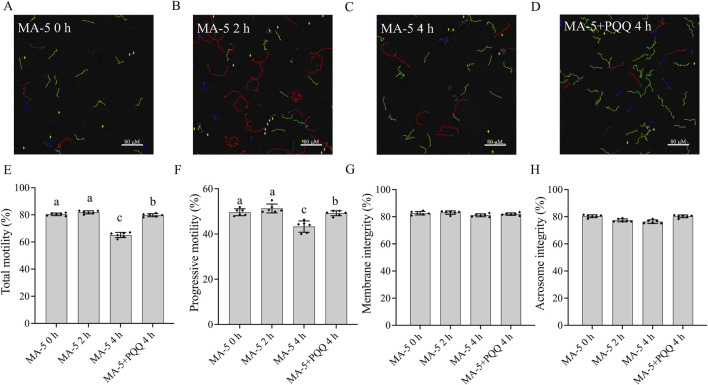
Effect of adding PQQ and MA-5 on boar sperm motility, membrane integrity, and acrosome integrity during incubation at 37°C. **(A–F)** Sperm motility was assessed using computer-assisted sperm analysis (CASA) following incubation with MA-5 for 4 h (n = 6). **(A–D)** Changes in the motility tracks of sperm generated by the CASA system. Total motility **(E)** and progressive motility **(F)** of boar sperm incubated with MA-5 or MA-5 plus PQQ (n = 6). Membrane integrity **(G)** and acrosome integrity **(H)** of sperm treated with 1 nM MA-5 or 1 nM MA-5 plus 10 nM PQQ (n = 6). Values are specified as means ± standard deviation (SD). Columns with different lowercase letters indicate significant differences (p < 0.05). Bars = 80 μm. PQQ: pyrroloquinoline quinone.

CASA system-generated sperm tracks show that the combined addition of 10 nM PQQ and 1 nM MA-5 to the extender significantly (p < 0.05) improved boar sperm motility after 4 h of incubation (Figures 4A–D; Supplementary Figure S9). Moreover, MA-5 treatment without PQQ showed lower values for sperm motility similar to that of the control at 4 h of incubation (Figure 4E, p < 0.05). Furthermore, the addition of 10 nM PQQ increased sperm progressive motility compared to that of the control after 4 h of incubation (Figure 4F, p < 0.05). In addition, sperm membrane integrity and acrosome integrity were not significantly changed (p > 0.05) with the addition of MA-5 with and without the addition of 10 nM PQQ at 4 h of incubation (Figures 4G, H).

**FIGURE 5:**
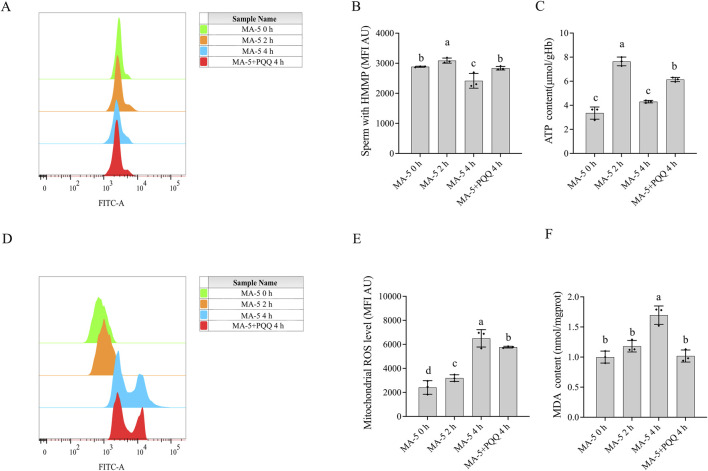
Effect of adding PQQ and MA-5 on boar sperm mitochondrial membrane potential, ATP levels, mitochondrial ROS, and MDA levels. **(A, B)**: Flow cytometry analysis of mitochondrial membrane potential in boar sperm after treatment with 1 nM MA-5 or 1 nM MA-5 plus 10 nM PQQ. **(C)**: Intracellular ATP levels in sperm after treatment with 1 nM MA-5 or 1 nM MA-5 plus 10 nM PQQ. **(D,E)**: Flow cytometric analysis of mitochondrial ROS levels in boar sperm after treatment with 1 nM MA-5 or 1 nM MA-5 plus 10 nM PQQ. **(F)**: Malondialdehyde (MDA) levels in boar sperm after treatment with 1 nM MA-5 or 1 nM MA-5 plus 10 nM PQQ. Values are specified as means ± standard deviation (SD). Columns with different lowercase letters indicate significant differences (p < 0.05) (n = 3).

To maintain mitochondrial membrane potential and sperm function, mitigating excessive ROS production over prolonged periods is crucial. However, the mitochondrial membrane potential decreased despite supplementation with MA-5 at 4 h due to the accumulation of ROS. Conversely, the addition of 10 nM PQQ treatment and MA-5 at the same time point led to a significant increase (p < 0.05) in mitochondrial membrane potential and reduced ROS level compared to that in the control (Figures 5A, B, D, E). Therefore, the addition of 10 nM PQQ restored mitochondrial function by reducing ROS levels.

Interestingly, at the 2-h time point of incubation, an increase in MA-5 ATP levels was observed, but the increase was not sustained at 4 h of incubation due to weakened mitochondrial function. Consequently, adding 10 nM PQQ at 4 h enhanced mitochondrial activity and increased ATP content (Figure 5C, p < 0.05). Furthermore, the addition of 10 nM PQQ treatment significantly reduced MDA content compared to that in the control after 4 h of incubation (Figure 5F, p < 0.05).

### 3.5 Addition of PQQ enhanced mitochondrial transcription and translation at 4 h

**FIGURE 6:**
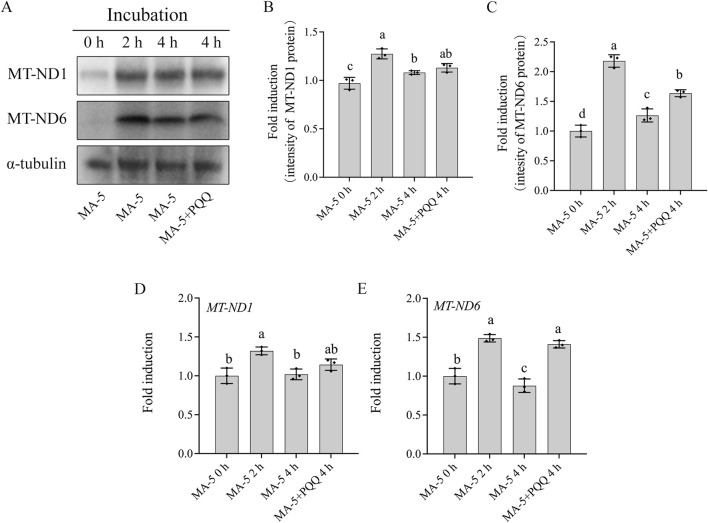
Addition of PQQ and MA-5 improves the expression of MT-ND1 and MT-ND6 in boar sperm. **(A–C)** Western blotting analysis of MT-ND1 and MT-ND6 in boar sperm after treatment with 1 nM MA-5 or 1 nM MA-5 plus 10 nM PQQ. **(D, E)** Expression of *MT-ND1* and *MT-ND6* genes in boar sperm after treatment with 1 nM MA-5 or 1Nm MA-5 plus 10 nM PQQ. Values are specified as means ± standard deviation (SD). Columns with different lowercase letters indicate significant differences (p < 0.05) (n = 3).

Mitochondrial-encoded proteins such as MT-ND1 and MT-ND6 are essential for sperm mitochondrial function. The expression of these mitochondrial proteins in boar sperm was analyzed in 1 nM MA-5 treatment (the control) and 1 nM MA-5 plus 10 nM PQQ treatment groups after up to 4 h of incubation. Results indicate that the addition of 10 nM PQQ significantly increased (p < 0.05) the expressions of MT-ND6 and MT-ND1 proteins compared to MA-5 treatment in the BTS medium after 4 h at 37°C (Figures 6A–C; Supplementary Figure S5). Similarly, PQQ treatment significantly increased the expression levels of *MT-ND6* and *MT-ND1* genes compared to the control in the BTS medium after 4 h at 37°C (Figures 6D,E, p < 0.05).

**FIGURE 7:**
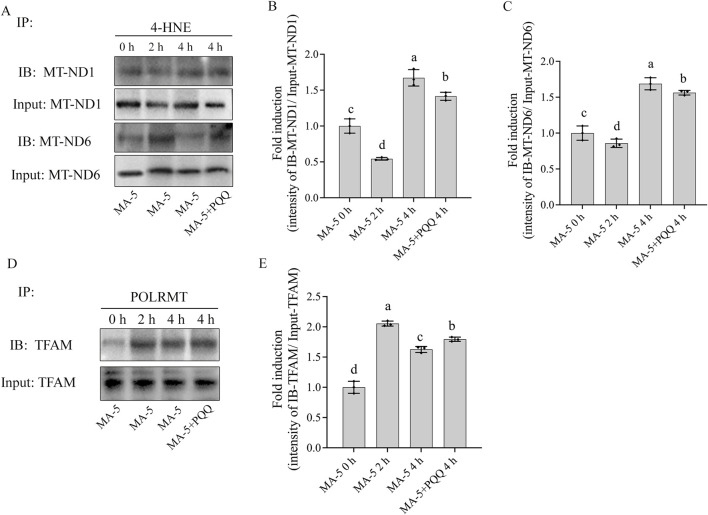
Effects of MA-5 and PQQ on 4-HNE-modified mitochondrial proteins in boar sperm. **(A)** Immunoprecipitates were immunoblotted (IB) with MT-ND1 and MT-ND6 antibody. **(B,C)** Quantitative expression of IB-MT-ND1 and IB-MT-ND6 over the input generated from Western blotting. **(D)** Immunoprecipitates were immunoblotted (IB) with TFAM antibody. **(E)** Quantitative expression of IB-TFAM over the input generated from Western blotting. Values are specified as means ± standard deviation (SD). Columns with different lowercase letters indicate significant differences (p < 0.05) (n = 3).

**FIGURE 8:**
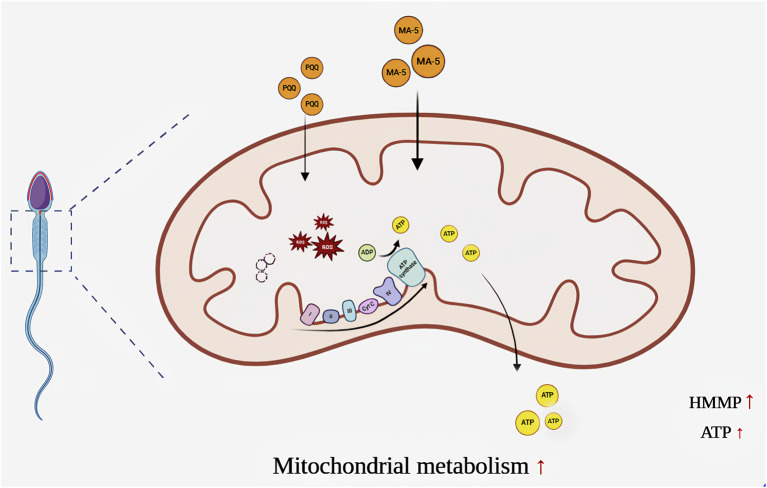
Adding mitochondrial acid 5 accelerated mitochondrial transcription and translation in sperm, thereby increasing the expression of sperm mitochondrial proteins. This led to an increase in ATP content and mitochondrial activity and then to an increase in sperm motility. The Figure was made with BioRender.com.

In addition, during the sperm incubation with MA-5 for 4 h, it was observed that the levels of 4-hydroxynonenal (4-HNE) modified MT-ND1 and MT-ND6 proteins in boar sperm at the 4-h time point of incubation were significantly higher than those in the 0-h or 2-h time points of incubation, and the addition of 10 nM PQQ to the MA-5 treatment downregulated the expression levels of the 4-HNE-modified MT-ND1 and MT-ND6 proteins after 4 h of incubation (Figures 7A–C; Supplementary Figure S6). In addition, the POLRMT–TFAM complex was significantly increased during the 4 h of incubation; interestingly, the values of the POLRMT–TFAM complex at the 4-h time point of incubation was lower than those in the 2-h time point of incubation, but the addition of 10 nM PQQ increased the level of the POLRMT–TFAM complex compared to that in the MA-5 treatment at the 4-h time point of incubation (Figures 7D, E; Supplementary Figure S7). Therefore, the addition of 10 nM PQQ restored mitochondrial transcription and translation in the mitochondria by reducing ROS levels. Thus, this study shows that PQQ supplementation helps mitigate the reduction of mitochondrial transcription proteins induced by excessive ROS (Figures 7, 8).

## 4 Discussion

Mitochondria are essential organelles that play a central role in energy production, calcium and lipid homeostasis, apoptosis regulation, and redox balance in mammalian cells (Liu et al., 1996; Bratic and Trifunovic, 2010; Labbé et al., 2014). In somatic cells, mitochondrial function is tightly regulated by the turnover and modification of mitochondrial proteins, which are essential for maintaining oxidative homeostasis and metabolic activity (Marchi et al., 2012; Youle and van der Bliek, 2012; Golpich et al., 2017). In sperm, mitochondrial activity is directly related to motility and fertilization capacity, making the maintenance of mitochondrial function particularly crucial during storage or incubation.

Previous studies demonstrated that the addition of MA-5 significantly enhanced mitochondrial metabolism and ATP production in various cell types (Lowell and Shulman, 2005). Consistent with this, in our previous study, supplementation with MA-5 in ram sperm extender increased mitochondrial protein expression, including MT-ND1 and MT-ND6, during storage at 4°C (Wang et al., 2024). Similarly, in the present study, addition of 1 nM MA-5 to boar sperm maintained mitochondrial gene expression and increased the TFAM–POLRMT complex level, indicating its potential role in preserving mitochondrial transcriptional activity during storage. Although the level of the TFAM–POLRMT complex was improved by supplementation of MA-5, whether the level of TFAM in boar sperm changed by MA-5 supplementation or not is unclear, as we did not analyze the TFAM expression. It is one of the limitations in the present study. Further study is needed to elucidate how MA-5 activates sperm mitochondria in future.

Mitochondrial-encoded genes, such as those encoding components of the electron transport chain (ETC), play a vital role in ATP generation *via* oxidative phosphorylation (OXPHOS). Specifically, 13 protein subunits of the ETC are encoded by mtDNA, and their expression contributes to sperm motility, particularly progressive and linear movement (Chomyn et al., 1985; Amaral et al., 2013; Zhu et al., 2019b). Our data revealed that MA-5 enhanced mitochondrial function by increasing mitochondrial membrane potential (MMP), which is a critical driving force for ATP synthesis (Baumber et al., 2000; Maranzana et al., 2013). Supplementation with 1 nM of MA-5 significantly increased the ATP content, suggesting that MA-5 positively modulates mitochondrial OXPHOS efficiency.

Despite the positive effects of MA-5, oxidative stress remains a major challenge during sperm incubation. Excessive ROS accumulation reduces mitochondrial protein expression (MT-ND1 and MT-ND6) and impairs mitochondrial transcription machinery (TFAM and POLRMT) (Xie et al., 2012; Maranzana et al., 2013). In our study, prolonged incubation (4 h at 37°C) led to decreased sperm motility and ATP levels and increased ROS generation. It was due to the excessive ROS generated as a by-product by the sperm’s high mitochondrial membrane potentials, activated by MA-5 supplementation. Moreover, supplementation of PQQ to the MA-5 treatment helps mitigate ROS damage. However, we did not incubate sperm with PQQ only as a control in the present study. Whether the reduction in ROS damage of boar sperm is due solely to PQQ supplementation or the combination with MA-5 is still unclear, and further study is needed to confirm it. PQQ has been shown to exhibit superior antioxidant properties compared to conventional antioxidants such as GSH and Trolox (Jia et al., 2015). Unlike GSH and Trolox, which primarily function in the cytoplasm and exhibit limited efficacy under prolonged oxidative stress (Flores et al., 2023; Vilchis-Landeros et al., 2024), PQQ can undergo continuous redox cycling without degradation, providing sustained mitochondrial protection (Jonscher et al., 2021). Beyond ROS scavenging, PQQ promotes mitochondrial biogenesis by activating signaling pathways such as PGC-1α and NRF1, which are not typically influenced by classical antioxidants (Sangha et al., 2023; Liu et al., 2024). Additionally, PQQ upregulates the expression levels of endogenous antioxidant enzymes, including SOD, CAT, and GPX, offering both direct and indirect protection against oxidative stress (Jonscher et al., 2021). In our findings, PQQ supplementation to the MA-5 treatment restored mitochondrial activity and ATP content, thereby improving sperm motility. Although ROS participates in capacitation and acrosome reaction (O’Flaherty et al., 2006), no changes in capacitation status or non-progressive motility were detected, indicating that decreased motility is primarily due to oxidative damage rather than functional activation.

However, in the present study, when boar sperm were incubated with different doses of MA-5 (0.01, 0.1, 1, and 10 nM) for 4 h, it was found that the addition of 10 nM MA-5 significantly decreased the sperm membrane integrity compared to that of the control; meanwhile, the other treatments showed no significant difference. This indicates that the reduction in sperm quality through 10 nM MA-5 treatment might be due to the toxicity induced by high concentrations of MA-5. In terms of the effect on the sperm quality, although the 1 nM MA-5 treatment showed the best positive effect among all the treatments, there might be another optimal dose of MA-5 for boar sperm, between 1 nM and 10 nM; unfortunately, we did not perform the dose–response curve with intermediate points from 1 nM to 10 nM, which is the limitation of this study. Further research is required to explore the optimal dosage of MA-5 on boar sperm during storage.

## 5 Conclusion

As shown in Figure 8, MA-5 supplementation increases mitochondrial function and sperm quality by increasing ATP production. However, this process increases ROS and MDA content in sperm mitochondria after 4 h of incubation. PQQ, a mitochondrial target antioxidant, helps reduce the ROS level. Combined treatment with 1 nM MA-5 and 10 nM PQQ resulted in much higher improvement in sperm quality and mitochondrial function. These findings highlight the potential of MA-5 and PQQ as effective agents for improving sperm quality during storage and incubation. Further research is required to explore the long-term effects and optimal dosage of MA-5 in boar sperm for use in AI practices.

## Data Availability

The original contributions presented in the study are included in the article/Supplementary Material; further inquiries can be directed to the corresponding author.
