# Supplementary material for: Mitochonic acid 5 increases boar sperm quality by mitigating mitochondrial dysfunction
Source: Front Cell Dev Biol. 2025 Jun 16;13:1583951. doi: 10.3389/fcell.2025.1583951 (PMC12206791; doi:10.3389/fcell.2025.1583951)
Supplement: Supplementary file 1 [file DataSheet1.docx]

Supplementary Figures and legends

**
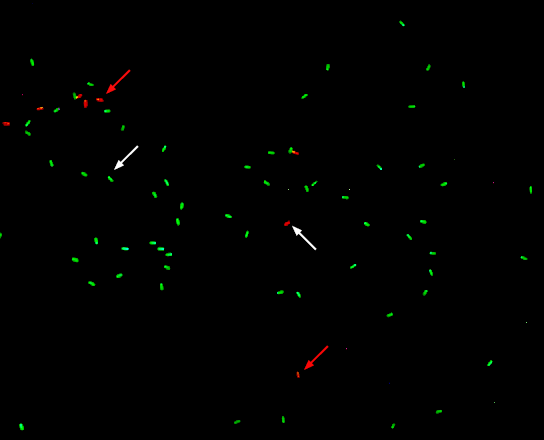
**

**Supplementary Figure 1.** Effect of different concentrations of MA-5 on sperm membrane integrity. The procedure led to the selective labelling of dead (plasma membrane damage, red arrow) and live (intact, white arrow) sperm cells with red or green fluorescence, respectively.

**
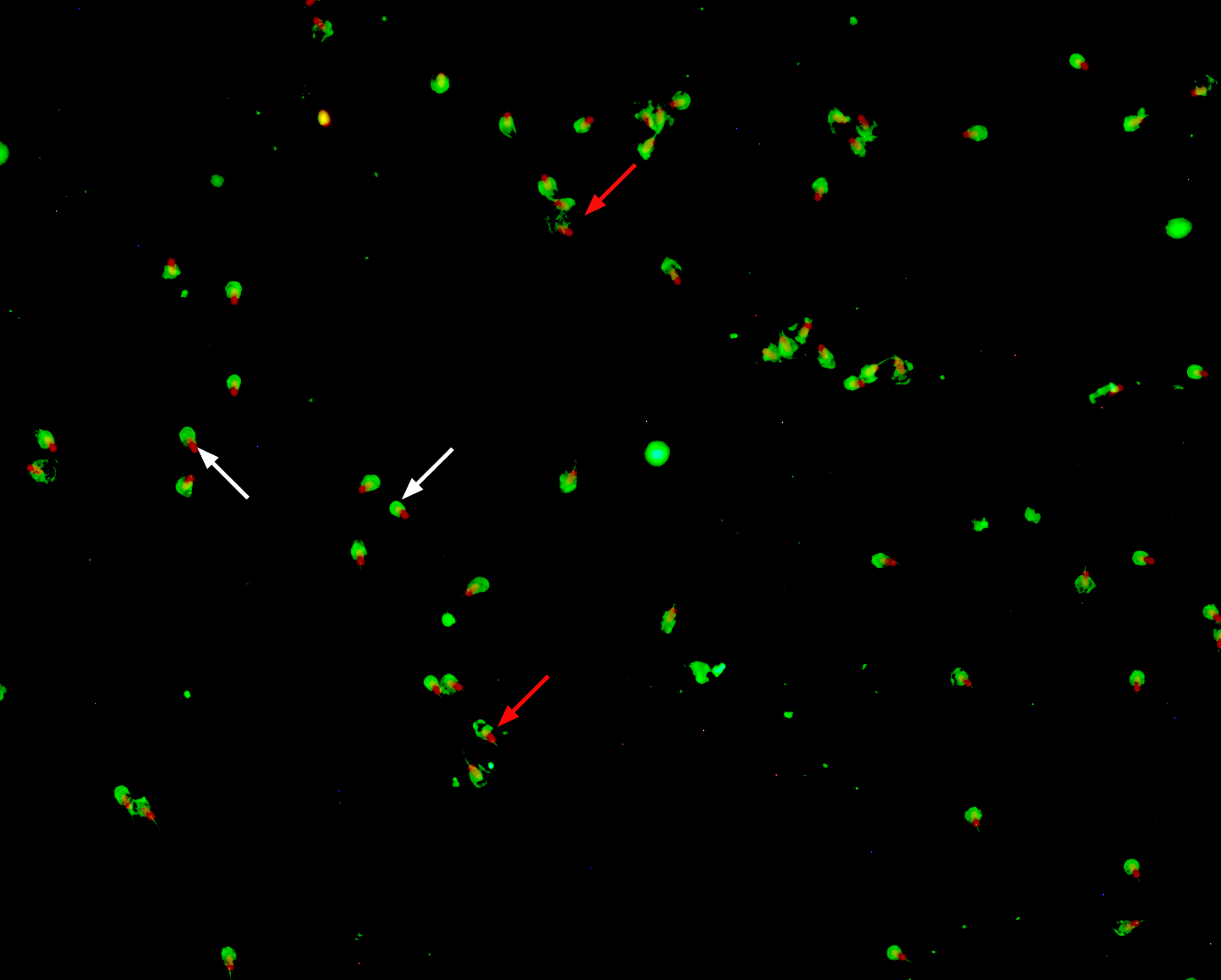
**

**Supplementary Figure 2.** Effect of different concentrations of MA-5 on sperm acrosome integrity. There are two groups of sperm after staining with FITC-PNA/PI: damaged acrosome (red arrow) and intact acrosome (white arrow).

**
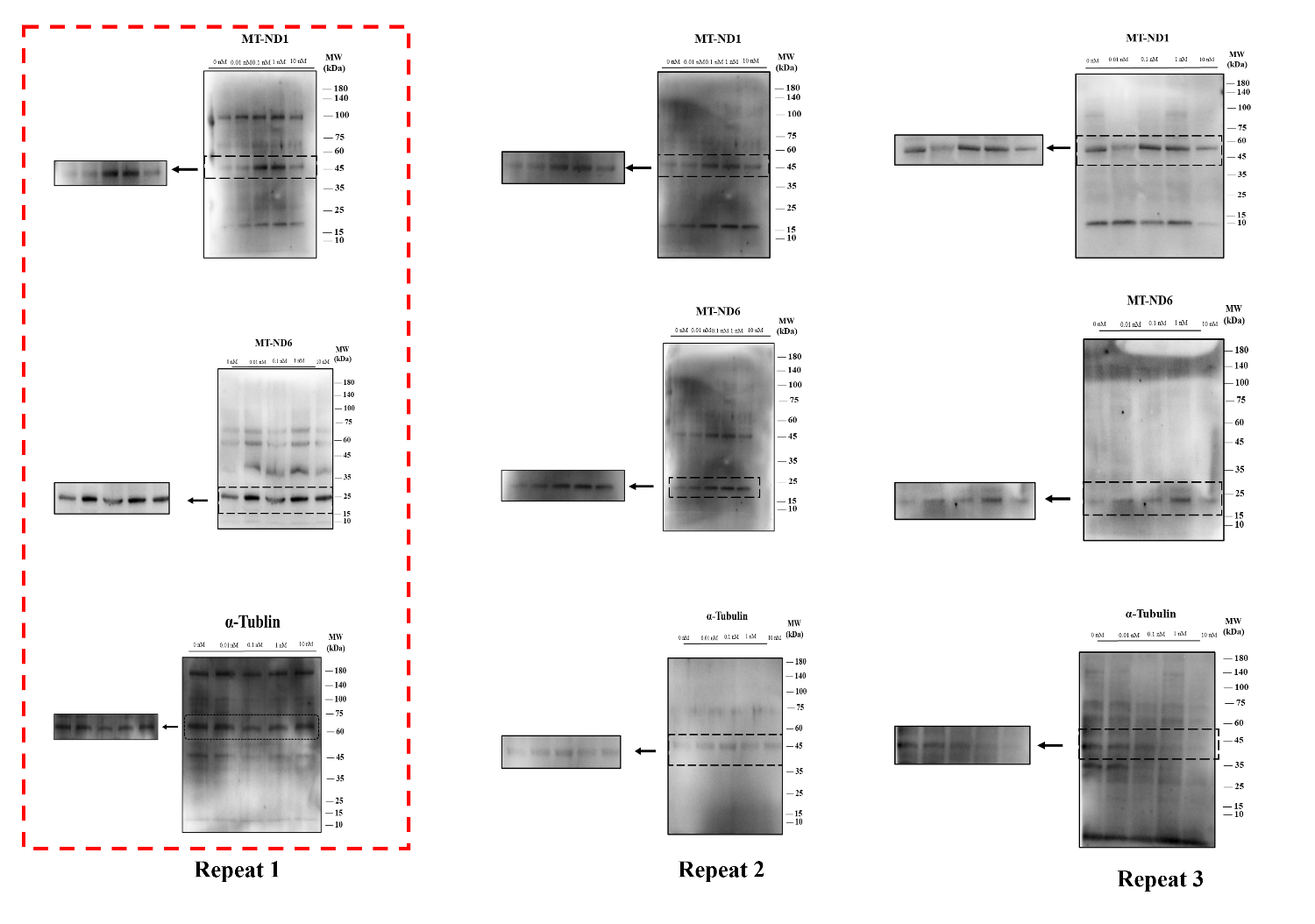
**

**Supplementary Figure 3.** Effect of different concentrations of MA-5 on the expression of mitochondrial proteins (MT-ND1, MT-ND6) in boar sperm.


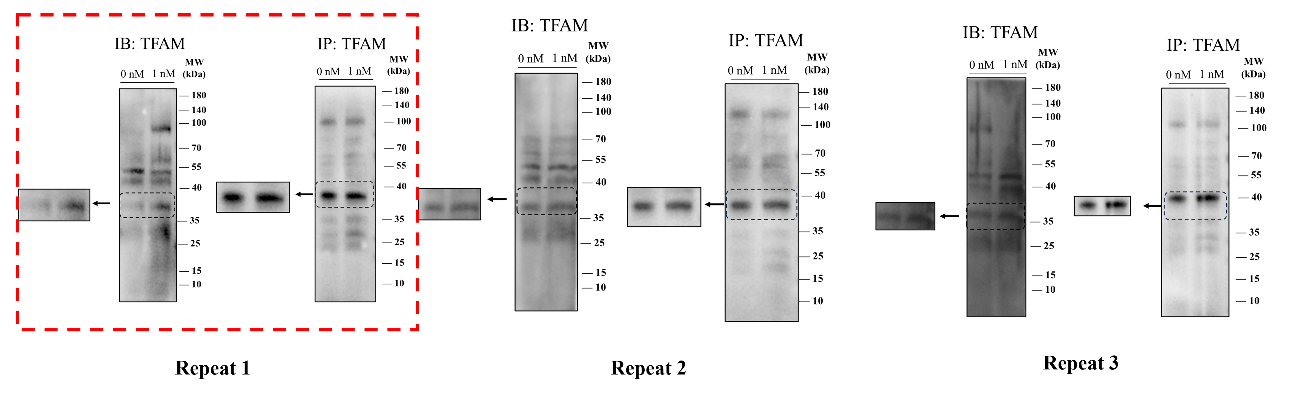


**Supplementary Figure 4**. The immunoprecipitates were immunoblotted (IB) with TFAM antibody.

**
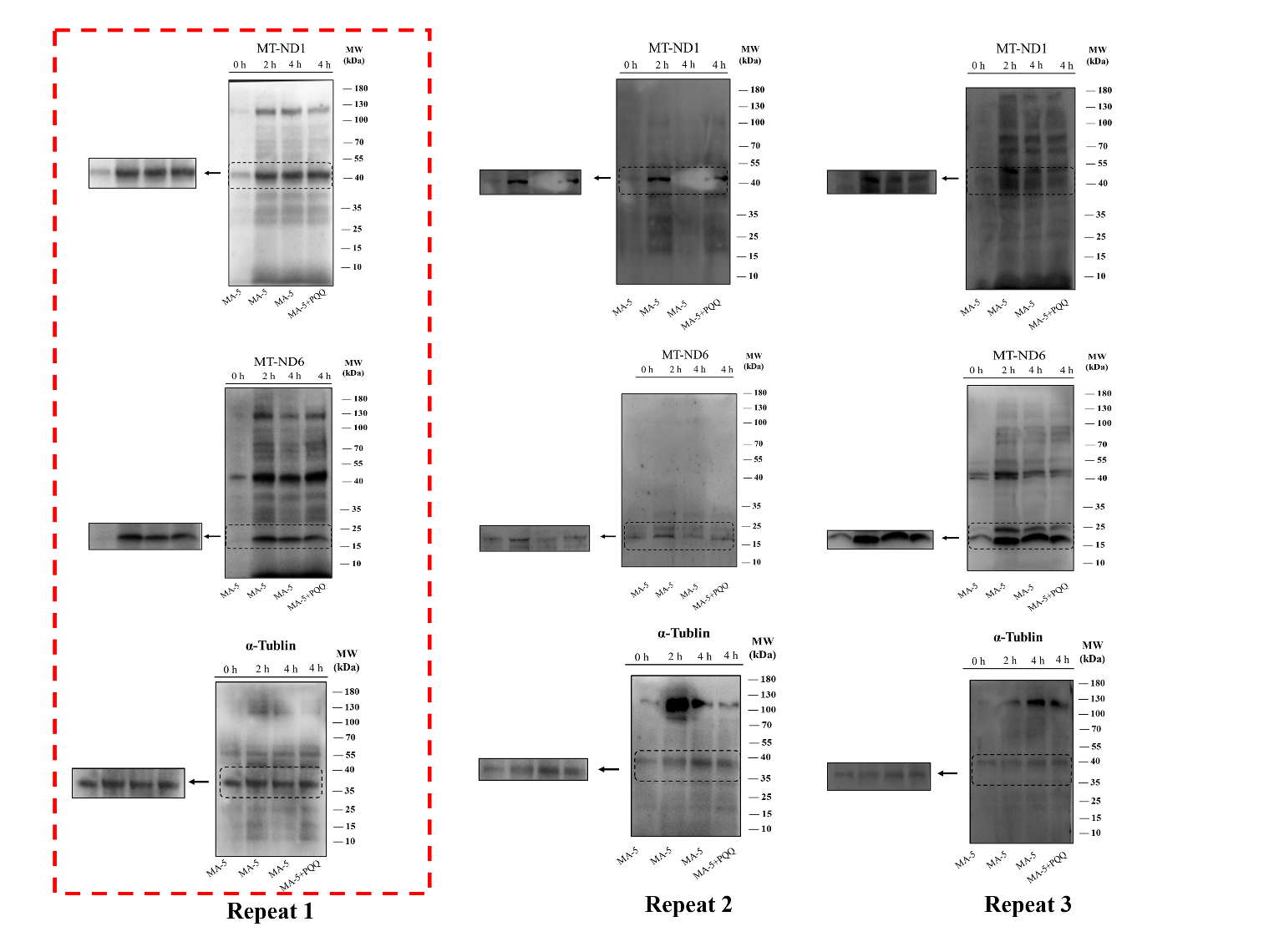
**

**Supplementary Figure 5**. Effect of 1 nM MA-5 and 10 nM PQQ on the expression of mitochondrial proteins (MT-ND1, MT-ND6) in boar sperm.

**
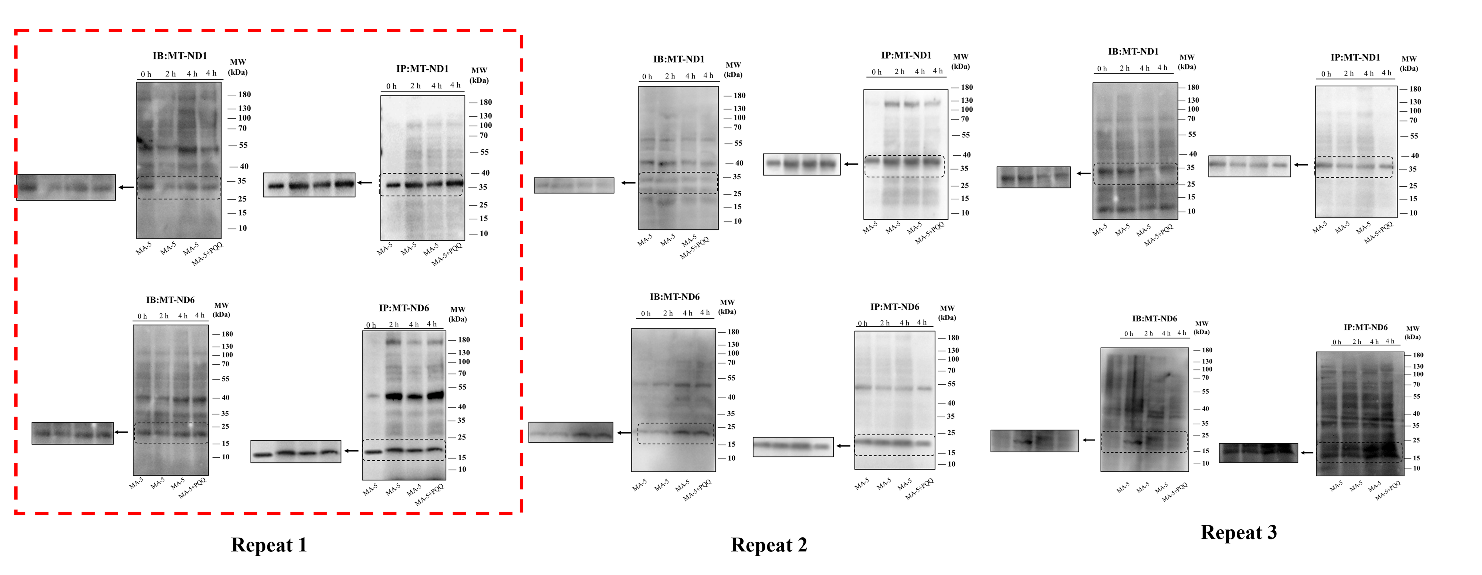
**

**Supplementary Figure 6**. The immunoprecipitates were immunoblotted (IB) with MT-ND1 and MT-ND6 antibodies.


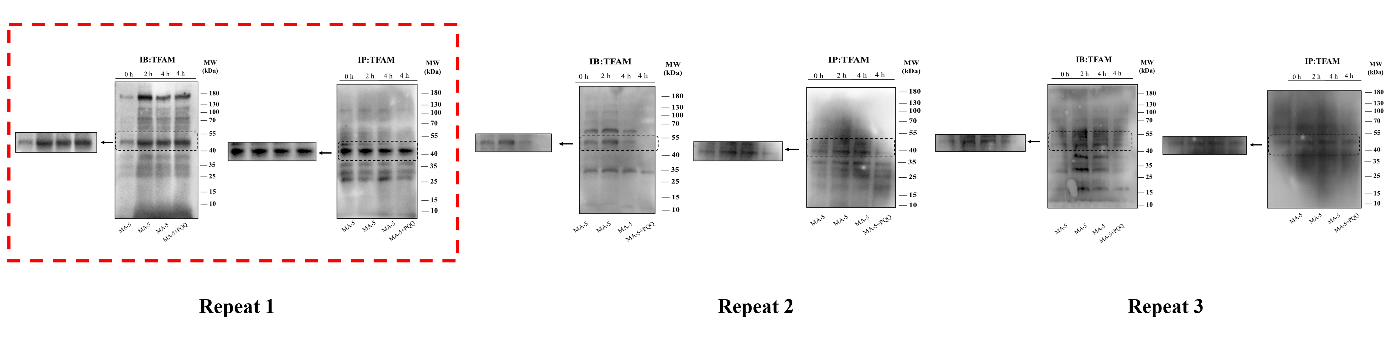


**Supplementary Figure 7.** The immunoprecipitates were immunoblotted (IB) with TFAM antibody after the sperm were incubated with MA-5 and PQQ.


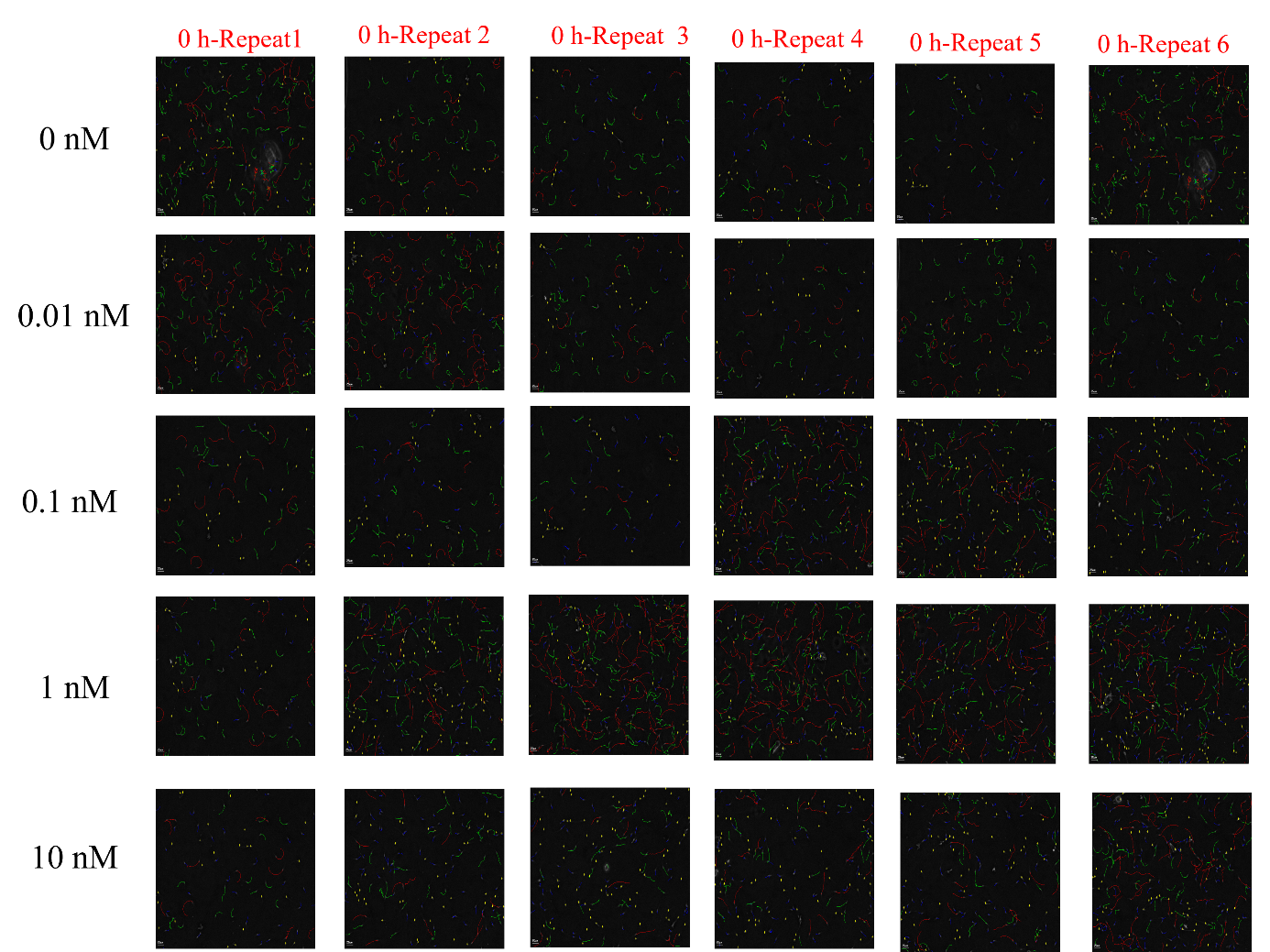


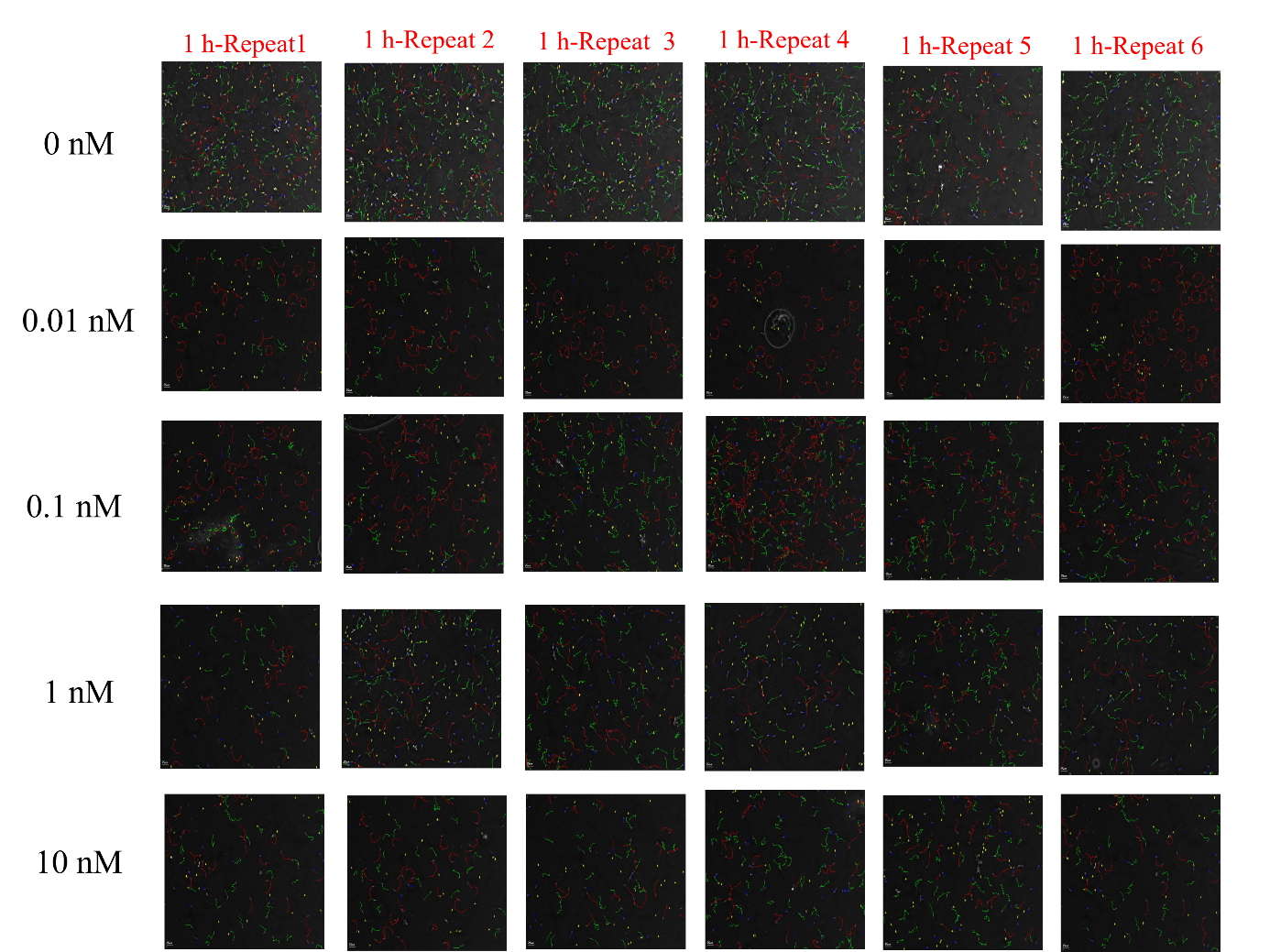


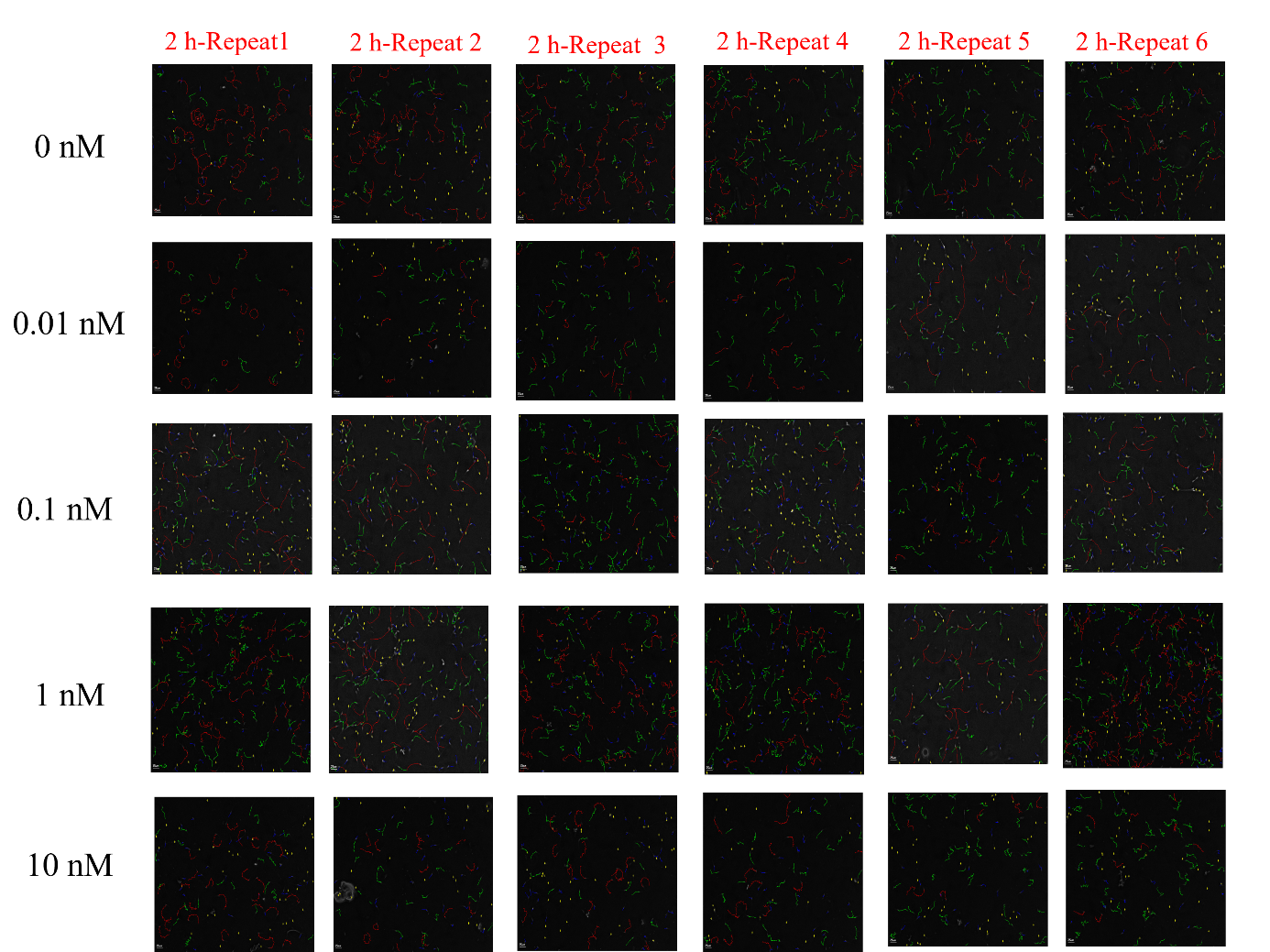


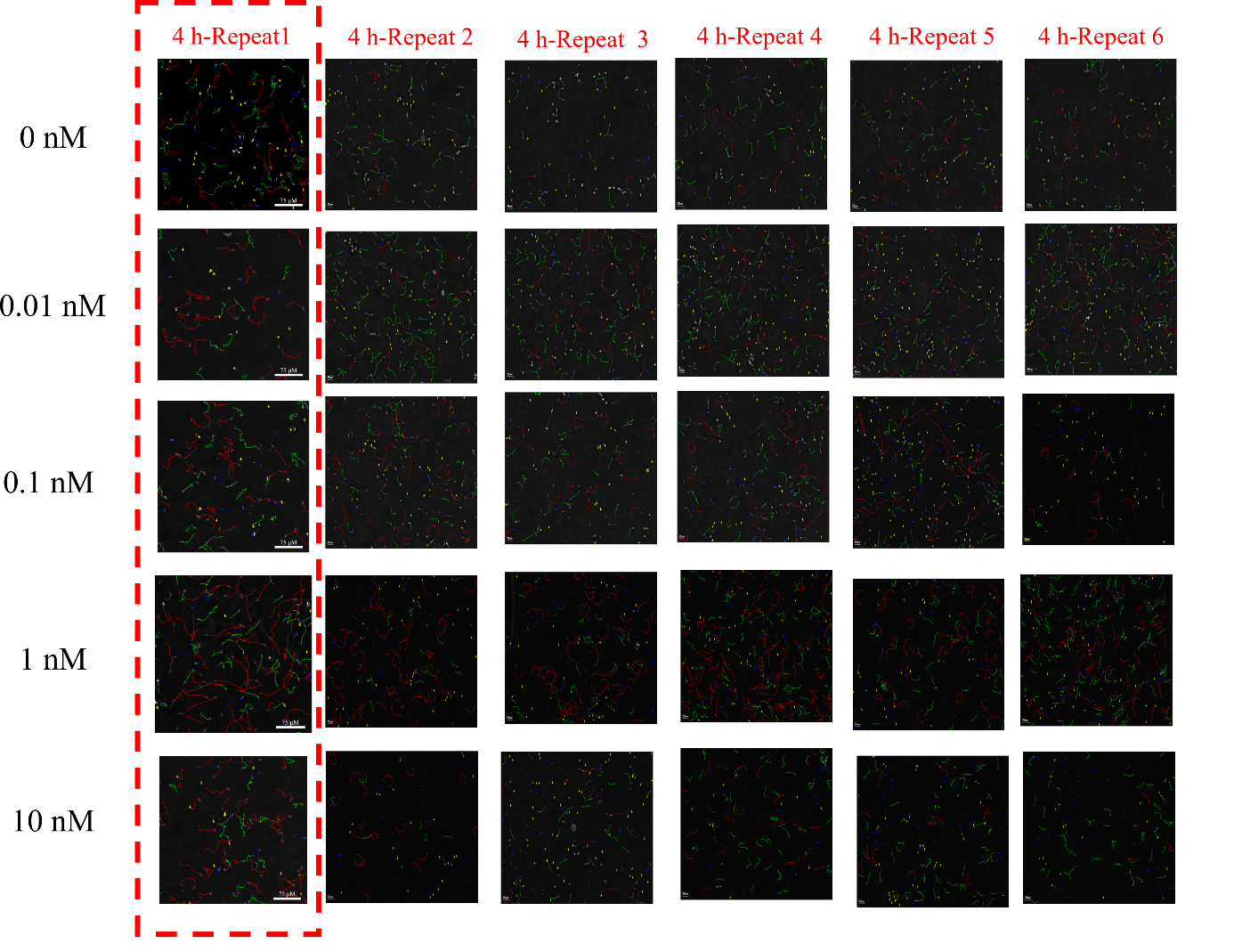


**Supplementary Figure 8.** Effect of different concentrations of MA-5 on sperm motility during 4 h of incubation at 37 °C.

**
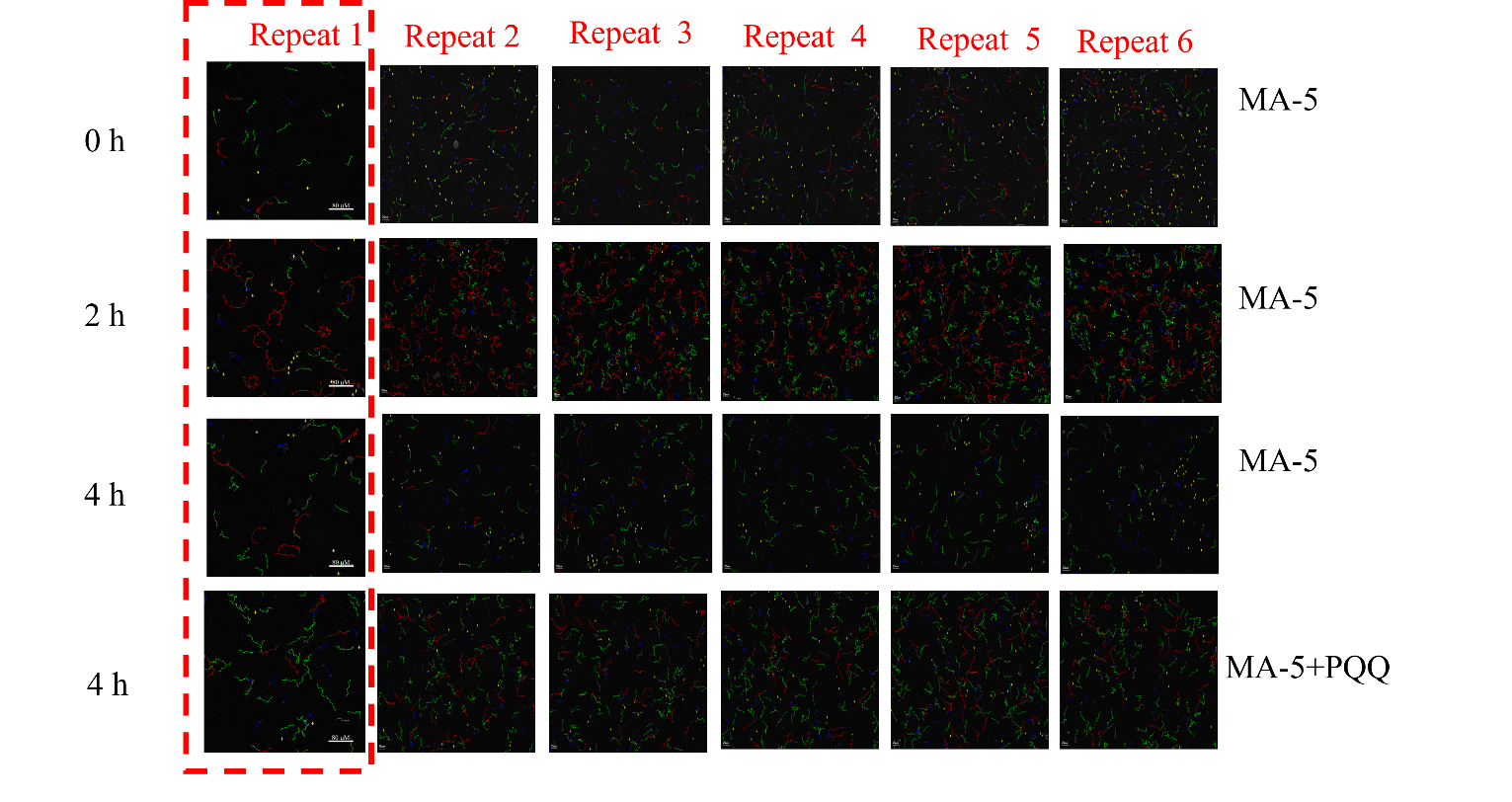
**

**Supplementary Figure 9.** Effect of MA-5 or MA-5 + PQQ on boar sperm motility during 4 h of incubation at 37 °C.
